# Supplementary material for: Assessing knowledge and attitudes toward epilepsy among schoolteachers and students: Implications for inclusion and safety in the educational system
Source: PLoS One. 2021 Apr 2;16(4):e0249681. doi: 10.1371/journal.pone.0249681 (PMC8018618; doi:10.1371/journal.pone.0249681)
Supplement: S1 File — (DOCX) [file pone.0249681.s001.docx]

**Assessing knowledge and attitudes toward epilepsy among school-teachers and students in Southern Italy: a cross-sectional study**

Luigi Francesco Iannone^1^, Roberta Roberti^1^, Gabriele Arena^1^, Simone Mammone^1^, Patrizia Pulitano^2^, Giovambattista De Sarro^1^, Oriano Mecarelli^2^, Emilio Russo^1, *^

*^1^Science of Health Department, University Magna Graecia, Catanzaro, Italy; ^2^Human Neurosciences Department, Sapienza University, Rome, Italy.*

**Running Title:** Epilepsy stigma in educational setting

| **Table S1.** School life-related attitudes in teachers (n=667). | |
| --- | --- |
|  | **n (%)** |
| 1. *Have you ever had children with epilepsy in your classroom?* |  |
| Yes | 313 (46.8) |
| *Whether Yes, how many?* |  |
| 1 | 247 (37.0) |
| 2 | 47 (7.0) |
| 3 | 10 (1.5) |
| >3 | 9 (1.3) |
| 1. *How often have you been informed by parents of the form of epilepsy their child has?* |  |
| Always | 158 (50.5) |
| Only in some cases | 124 (39.6) |
| Never | 31 (9.9) |
| 1. *Do you know how to manage a child experiencing an epileptic attack?* |  |
| Very well | 98 (14.7) |
| Moderately | 252 (37.5) |
| Poorly | 236 (35.4) |
| No at all | 81 (12.1) |
| 1. *In the case of a seizure in class (with loss of consciousness, drop, and spams to the whole body) what would you do? ^a^* |  |
| Call an ambulance | 343 (51.4) |
| Have the person lie down on the ground and wait until the end of the attack | 306 (45.9) |
| Place something in the subject’s mouth | 278 (41.6) |
| Block the spasms of the limbs | 45 (6.7) |
| Administer medications endo-rectally | 190 (28.4) |
| Would not know what to do | 33 (4.9) |
| 1. *In your school are there difficulties in administering antiepileptic drugs during school-time?* |  |
| Yes | 144 (21.6) |
| No | 262 (39.3) |
| Don’t’ know | 261 (39.1) |
| 1. *In your opinion, to what extent does epilepsy impair children’s learning?* |  |
| Strongly | 38 (5.7) |
| Moderately | 197 (29.5) |
| Scarcely | 135 (20.2) |
| Not at all | 201 (30.1) |
| Do not know | 96 (14.4) |
| 1. *In your opinion, to what extent do children with epilepsy require support in school?* |  |
| Strongly | 134 (20.1) |
| Moderately | 238 (35.7) |
| Scarcely | 97 (14.5) |
| Not at all | 132 (19.8) |
| Do not know | 66 (9.9) |
| 1. *To what extent do children with epilepsy have mental and/or behaviour alterations?* |  |
| Strongly | 32 (4.8) |
| Moderately | 183 (27.4) |
| Scarcely | 135 (20.2) |
| Not at all | 183 (27.4) |
| Do not know | 134 (20.1) |
| 1. *In your opinion, to what extent do antiepileptic drugs affect learning and behaviour?* |  |
| Strongly | 60 (9.0) |
| Moderately | 216 (32.4) |
| Scarcely | 122 (18.3) |
| Not at all | 110 (16.5) |
| Do not know | 159 (23.8) |
| 1. *Based on your experience, how do classmates behave toward a child with epilepsy?* |  |
| Normally | 298 (44.7) |
| Try to help | 200 (30.0) |
| Tend to marginalize | 6 (0.9) |
| Do not know | 163 (24.4) |
| 1. *Compared with their healthy classmates, how should children with epilepsy be treated with respect to attitudes and demands?* |  |
| The same | 612 (91.8) |
| Differentiate | 32 (4.8) |
| Do not know | 23 (3.4) |
| 1. *In your experience, recreational and sports activity of the child with epilepsy must be:* |  |
| Normal | 524 (78.6) |
| Limited | 48 (7.2) |
| Do not know | 95 (14.2) |
| 1. *Which of the following sports do you think should not be recommended for a child with epilepsy? ^a^* |  |
| Soccer | 73 (10.9) |
| Tennis | 23 (3.4) |
| Swimming | 202 (30.3) |
| Skiing | 135 (20.2) |
| Athletics | 39 (5.8) |
| Boxing | 445 (66.7) |
| Cycling | 129 (19.3) |
|  |  |
| ^a^ *Multiple answer allowed.* | |

| **Table S2.** Attitudes toward social and individuals’ implications of epilepsy in teachers (n=667). | | |
| --- | --- | --- |
|  | **N (%)** | |
| 1. *To what extend does epilepsy limit marriage?* | |  |
| Strongly | | 21 (3.1) |
| Moderately | | 157 (23.5) |
| Scarcely | | 86 (12.9) |
| Not at all | | 266 (39.9) |
| Do not know | | 137 (20.5) |
| 1. *To what extent does epilepsy limit have children?* | |  |
| Strongly | | 46 (6.9) |
| Moderately | | 143 (21.4) |
| Scarcely | | 75 (11.2) |
| Not at all | | 241 (36.1) |
| Do not know | | 162 (24.3) |
| 1. *To what extent does epilepsy limit regular employment?* | |  |
| Strongly | | 73 (10.9) |
| Moderately | | 301 (45.1) |
| Scarcely | | 149 (22.3) |
| Not at all | | 81 (12.1) |
| Do not know | | 63 (9.4) |
| 1. *To what extent does epilepsy limit driving?* | |  |
| Strongly | | 288 (43.2) |
| Moderately | | 217 (32.5) |
| Scarcely | | 59 (8.8) |
| Not at all | | 29 (4.3) |
| Do not know | | 74 (11.1) |
| 1. *To what extent does epilepsy limit sports and leisure activities?* | |  |
| Strongly | | 36 (5.4) |
| Moderately | | 295 (44.2) |
| Scarcely | | 151 (22.6) |
| Not at all | | 122 (18.3) |
| Do not know | | 63 (9.4) |

| **Table S3.** Attitudes toward social and individuals’ implications of epilepsy in students (n=672). | |
| --- | --- |
|  | **N (%)** |
| *9. Do you think epilepsy is an important impediment for ^a^:* |  |
| Driving | 589 (87.6) |
| Job | 455 (67.7) |
| Sports | 393 (58.5) |
| Marriage/having children | 252 (37.5) |
| 10. *In the case of a seizure in class (with loss of consciousness, drop and spasms of the whole body) what would you do? ^a^* |  |
| Call an ambulance | 332 (49.4) |
| Have the person lie down on the ground and wait until the end of the attack | 251 (37.4) |
| Place something in the child's mouth | 319 (47.5) |
| Block the spasms of the limbs | 131 (19.5) |
| Administer medications endo-rectally | 20 (3.0) |
| Would not know what to do | 117 (17.4) |
|  | |

| **Table S4.** Comparison of the results of the survey on another teacher’s population in Italy (2010) with the present results ^a^. | | | |  |
| --- | --- | --- | --- | --- |
|  | **2010** | **2019** | |  |
|  | n (%) | n (%) | |  |
| 1. *Do you know the disease called “epilepsy”?* |  |  | |  |
| Yes* | 598 (99.7) | 667 (99.7) | |  |
| 1. *Do you know epilepsy ^b^:* |  |  | |  |
| By hearsay | 241 (40.3) | 170 (25.5) | |  |
| Personal or familial experience | 202 (33.8) | 215 (32.2) | |  |
| Friends/acquaintances | 184 (30.8) | 96 (14.4) | |  |
| Doctor/scientific information | 261 (43.6) | 220 (32.9) | |  |
| 1. *Have you ever seen a seizure? ^b^* |  |  | |  |
| Yes, personally (home, public. classroom) | 331 (55.3) | 376 (55.3) | |  |
| Yes, TV/movies | 45 (7.5) | 133 (19.9) | |  |
| Never | 254 (42.5) | 230 (34.5) | |  |
| 1. *What is the approximate prevalence of epilepsy Italy?* |  |  | |  |
| About 1/100 *(correct answer)* | 203 (33.9) | 148 (22.2) | |  |
| 1. *What do you think causes epilepsy? ^b^* |  |  | |  |
| Hereditary disease | 329 (55.0) | 292 (43.8) | |  |
| Birth defect | 324 (54.2) | 246 (36.9) | |  |
| Viral infection | 121 (20.2) | 114 (17.1) | |  |
| Head injury | 220 (36.8) | 232 (34.8) | |  |
| Brain tumour | 175 (29.3) | 198 (29.7) | |  |
| Psychological/psychiatric disease | 118 (19.7) | 112 (16.8) | |  |
| 1. *What is the age of onset of epilepsy?* |  |  | |  |
| Childhood only | 221 (37.0) | 213 (31.9) | |  |
| All ages | 362 (60.5) | 349 (59.1) | |  |
| Do not know | 10 (1.7) | 56 (8.4) | |  |
| 1. *Do you think epilepsy is a form of psychiatric disease?* |  |  | |  |
| Yes | 65 (10.9) | 51 (7.6) | |  |
| No | 510 (85.3) | 523 (78.4) | |  |
| Do not know | 10 (3.8) | 93 (13.9) | |  |
| 1. *Do you think epilepsy is treatable with ^b^:* |  |  | |  |
| Specific drugs | 553 (92.5) | 603 (90.4) | |  |
| Neurosurgery | 63 (10.5) | 92 (13.8) | |  |
| Other methods/do not know | 108 (18.0) | 91 (13.6) | |  |
| 1. *Do you think epilepsy is a curable illness?* |  |  | |  |
| Yes | 243 (40.6) | 201 (30.1) | |  |
| No | 280 (46.8) | 293 (43.9) | |  |
| Do not know | 75 (12.5) | 173 (25.9) | |  |
| 1. *To what extent does epilepsy limit marriage?* |  |  | |  |
| Strongly/moderately | 197 (33.0) | 178 (26.6) | |  |
| 1. *To what extent does epilepsy limit having children?* |  |  | |  |
| Strongly/moderately | 147 (24.6) | 189 (28.3) | |  |
| 1. *To what extent does epilepsy limit regular employment?* |  |  | |  |
| Strongly/moderately | 237 (39.7) | 374 (56.0) | |  |
| 1. *To what extent does epilepsy limit sports and leisure activities?* |  |  | |  |
| Strongly/moderately | 196 (32.8) | 331 (49.8) | |  |
| 1. *To what extent does epilepsy limit driving?* |  |  | |  |
| Strongly/moderately | 438 (73.2) | 305 (75.7) | |  |
| 1. *Do you know how to manage a person experiencing an epileptic attack?* |  |  | |  |
| Yes | 201 (33.6) | 350 (52.2) | |  |
| 1. *In the case of a seizure in class (with loss of consciousness, drop, and spams to the whole body) what would you do? ^b^* |  |  | |  |
| Place something in the subject’s mouth | 346 (57.9) | 278 (41.6) | |  |
| Block the spasms of the limbs | 73 (12.2) | 45 (6.7) | |  |
| Administer medications endo-rectally | 46 (7.7) | 190 (28.4) | |  |
| 1. *In your school are there difficulties in administering antiepileptic drugs during school-time?* |  |  | |  |
| Yes | 305 (51.0) | 144 (21.6) | |  |
| 1. *In your opinion, to what extent do children with epilepsy require support in school?* |  |  | |  |
| Strongly/moderately | 218 (36.4) | 372 (55.8) | |  |
|  |  |  | |  |
| *^a^ Only the answers to the same questions are shown.*  *^b^ Multiple answers allowed.*  * *Subjects who answered “no” to first question “Do you know the disease called epilepsy?” were excluded.* | | |  | |

| **Table S5.** Comparison of the results of the survey with another student’s population in Italy (2007). | | |
| --- | --- | --- |
|  | **2007** | **2019** |
|  | n (%) | n (%) |
| *1. Do you know the disease called “epilepsy”?* |  |  |
| Yes* | 545 (91.0) | 672 (98.8) |
| *2. Do you know epilepsy:* |  |  |
| By hearsay | 218 (37.0) | 327 (48.7) |
| Personal or familial experience | 143 (24.0) | 96 (14.3) |
| Friends/acquaintances | / | 116 (17.3) |
| Medical interviews | 14 (2.0) | 45 (6.7) |
| Read scientific pamphlets | 14 (2.0) | 101 (15.0) |
| Participation in courses | / | 154 (22.9) |
| *3. Have you ever seen a seizure?* |  |  |
| Classroom | 131 (22.0) | 51 (7.6) |
| Public place | / | 115 (17.1) |
| Home | / | 51 (7.6) |
| TV/movies | 199 (33.0) | 217 (32.3) |
| Never | / | 281 (41.8) |
| *4. What is the approximate prevalence of epilepsy in Italy?* |  |  |
| 1/10 | 17 (3.0) | 25 (3.7) |
| 1/100 *(correct answer)* | 94 (16.0) | 114 (17.0) |
| 1/1.000 | 172 (29.0) | 203 (30.2) |
| 1/10.000 | 78 (13.0) | 216 (32.1) |
| 1/100.000 | 236 (40.0) | / |
| 1/1.000.000 | 17 (3.0) | 25 (3.7) |
| Do not know | 94 (16.0) | 114 (17.0) |
| *5. What do you think causes epilepsy*? *^a^* |  |  |
| Hereditary disease | 168 (28.0) | 248 (36.9) |
| Birth defect | 88 (15.0) | 197 (29.3) |
| Viral infection | 10 (2.0) | 117 (17.4) |
| Head injury | 116 (19.0) | 288 (42.9) |
| Brain tumor | 26 (4.0) | 213 (31.7) |
| Stress | / | / |
| *6. Do you think epilepsy is a curable illness?* |  |  |
| Yes | 202 (43.0) | 273 (40.6) |
| No | 80 (13.0) | 222 (33.0) |
| Do not know | 315 (53.0) | 177 (26.3) |
| *7. Do you think epilepsy is a form of psychiatric disease?* |  |  |
| Yes | 333 (56.0) | 178 (26.5) |
| *8. Do you think epilepsy is treatable with:* |  |  |
| Specific drugs | 351 (59.0) | 465 (69.2) |
| Neurosurgery | 44 (7.0) | 167 (24.9) |
| Other methods | 5 (1.0) | 99 (14.7) |
| *9. Do you think epilepsy is an important impediment for ^a^:* |  |  |
| Driving | 139 (23.0) | 589 (87.6) |
| Job | 332 (56.0) | 455 (67.7) |
| Sports | 32 (5.0) | 393 (58.5) |
| Marriage/having children | 69 (12.0) | 252 (37.5) |
|  | | |
| ^a^ *Multiple answer allowed.*  **Subjects who answered “no” to first question “Do you know the disease called epilepsy?” were excluded.* | | |
