# Supplementary material for: Assessing knowledge and attitudes toward epilepsy among schoolteachers and students: Implications for inclusion and safety in the educational system
Source: PLoS One. 2021 Apr 2;16(4):e0249681. doi: 10.1371/journal.pone.0249681 (PMC8018618; doi:10.1371/journal.pone.0249681)
Supplement: S1 Appendix — (DOCX) [file pone.0249681.s002.docx]

**S1 Appendix**. Teachers survey questionnaire (English translation)

***A. General and specific knowledge of epilepsy***

*1. Do you know the disease called “epilepsy”?*

Yes

No

*2. Do you know epilepsy:*

By hearsay

Personal or familial experience

Friends/acquaintances

Medical interviews

Read scientific pamphlets

Participation in training courses

*3. Have you ever seen a seizure?*

Classroom

Public place

Home

TV/movies

Never

*4. What is the approximate prevalence of epilepsy in Italy?*

1/10

1/100

1/1,000

1/10,000

1/100,000

1/1,000,000

Don't know

*5. What do you think causes epilepsy?*

Hereditary disease

Birth defect

Viral infection

Head injury

Brain tumor

Psychological disease

*6. What is the age of onset of epilepsy?*

Childhood

Adult

All ages

Don't know

*7. Do you think epilepsy is a form of psychiatric disease?*

Yes

No

Don't know

*8. Do you think epilepsy is treatable with:*

Specific drugs

Neurosurgery

Other methods

Don't know

*9. Do you think epilepsy is a curable illness?*

Yes

No

Don't know

***B. Personal and social implications***

*10. To what extent does epilepsy limit marriage?*

Strongly

Moderately

Scarcely

Not at all

Don't know

*11. To what extent does epilepsy limit having children?*

Strongly

Moderately

Scarcely

Not at all

Don't know

*12. To what extent does epilepsy limit regular employment?*

Strongly

Moderately

Scarcely

Not at all

Don't know

*13. To what extent does epilepsy limit driving?*

Strongly

Moderately

Scarcely

Not at all

Don't know

*14. To what extent does epilepsy limit sports and leisure activities?*

Strongly

Moderately

Scarcely

Not at all

Don't know

**C. School life-related attitudes**

*15. Have you ever had children with epilepsy in your classroom?*

Yes/No

Whether Yes, how many? 1 2 3 >3

*16. How often have you been informed by parents of the form of epilepsy their child has?*

Always

Only in some cases

Never

*17. Do you know how to manage a child experiencing an epileptic attack?*

Very well

Moderately

Poorly

Don't know

*18. In the case of a seizure in class (with loss of consciousness, drop, and spasms of the whole body) what would you do?*

Call an ambulance

Have the person lie down on the ground and wait until the end of

the attack

Place something in the child's mouth

Block the spasms of the limbs

Administer medications endorectally

Would not know what to do

*19. In your school are there difficulties in administering antiepileptic drugs during school hours?*

Yes

No

Don't know

*20. In your opinion, to what extent does epilepsy impair children's*

*learning?*

Strongly

Moderately

Scarcely

Not at all

Don't know

*21. In your opinion, to what extent do children with epilepsy require support in school?*

Strongly

Moderately

Scarcely

Not at all

Don't know

*22. To what extent do children with epilepsy have mental and/or behavior alterations?*

Strongly

Moderately

Scarcely

Not at all

Don't know

*23. In your opinion, to what extent do antiepileptic drugs affect learning and behavior?*

Strongly

Moderately

Scarcely

Not at all

Don't know

*24. In your opinion, to what extent do children with epilepsy have relationship problems with other children?*

Strongly

Moderately

Scarcely

Not at all

Don't know

*25. Compared with their healthy classmates, how should children with epilepsy be treated with respect to attitudes and demands?*

The same

Differently

Don't know

*26. Based on your experience, how do classmates behave toward a child with epilepsy?*

Normally

Try to help

Tend to marginalize

Don't know

*27. In your experience, recreational and sports activity of the child with epilepsy must be:*

Normal

Limited

Don't know

*28. Which of the following sports do you think should absolutely not be recommended for a child with epilepsy?*

Soccer

Tennis

Swimming

Skiing

Athletics

Boxing

Cycling

All

**S1 Appendix**. Teachers survey questionnaire (Original, Italian language)

***A. Conoscenza generale sull’epilessia***

*1. Conosci una malattia chiamata “Epilessia”?*

Si

No

*2. Come conosci l’epilessia:*

Per sentito dire

Da esperienze personali/familiari

Da amici/parenti

Da un medico o da personale sanitario

Da articoli scientifici

Da corsi di formazione/universitari

*3. Hai mai visto una crisi epilettica?*

Si, in classe/aula

Si, in un luogo pubblico

Si, a casa

Si, in tv/film

No, mai

*4. Quanto è diffusa l’epilessia in Italia?*

1/10

1/100

1/1,000

1/10,000

1/100,000

1/1,000,000

Non lo so

*5. Secondo te qual è la causa dell’epilessia?*

Malattia ereditaria

Difetti di nascita

Infezioni virali

Stress

Traumi alla testa

Tumori cerebrali

Disturbi psicologici/psichiatrici

*6. Qual è l’età di insorgenza dell’epilessia?*

Infanzia

Età adulta

A tutte le età

Non lo so

*7. Pensi che l'epilessia sia una forma di malattia psichiatrica? **

Si

No

Non lo so

*8. Pensi che l’epilessia sia curabile con:*

Farmaci specifici

Neurochirurgia

Altri metodi

Non lo so

*9. Pensi che l’epilessia sia una malattia da cui si può guarire?*

Si

No

Non lo so

***B. Implicazioni personali e sociali***

*10.  In che misura l'epilessia limita il matrimonio?*

Fortemente

Moderatamente

Appena

Per nulla

Non lo so

*11. In che misura l'epilessia limita l'avere figli?*

Fortemente

Moderatamente

Appena

Per nulla

Non lo so

*12. In che misura l'epilessia limita la vita lavorativa?*

Fortemente

Moderatamente

Appena

Per nulla

Non lo so

*13. In che misura l'epilessia limita la guida?*

Fortemente

Moderatamente

Appena

Per nulla

Non lo so

*14. In che misura l'epilessia limita gli sport e le attività del tempo libero?*

Fortemente

Moderatamente

Appena

Per nulla

Non lo so

**C. Comportamenti nell’ambiente scolastico**

*15. Hai mai avuto uno studente nella tua classe con epilessia?*

Si/No

Se si, quanti? 1 2 3 >3

*16. Quante volte sie stato informato dai genitori della forma di epilessia del loro bambino?*

Sempre

Solo in alcuni casi

Mai

*17. Sai come gestire un alunno che sta avendo un attacco epilettico?*

Si, molto bene

Si, ma non molto bene

Non in maniera sufficiente

No

*18. In presenza di un attacco epilettico (es. con perdita di conoscenza, caduta e spasmi di tutto il corpo) cosa faresti?*

Chiamo un'ambulanza

Faccio sdraiare la persona a terra e attendo la fine dell'attacco

Metto qualcosa in bocca, per evitare lesioni alla lingua

Blocco gli spasmi di braccia e gambe

Somministro farmaci per via rettale

Non saprei cosa fare

*19. Nella tua scuola ci sono difficoltà nella somministrazione dei farmaci antiepilettici durante l'orario scolastico?*

Si

No

Non lo so

*20. Secondo te, fino a che punto l'epilessia danneggia l'apprendimento degli alunni?*

Fortemente

Moderatamente

Appena

Per nulla

Non lo so

*21. Secondo te, in che misura i bambini con epilessia hanno bisogno di un supporto a scuola?*

Fortemente

Moderatamente

Appena

Per nulla

Non lo so

*22. In che misura i bambini con epilessia hanno alterazioni mentali e/o comportamentali?*

Fortemente

Moderatamente

Appena

Per nulla

Non lo so

*23. Secondo te, fino a che punto influiscono i farmaci antiepilettici sull'apprendimento e sul comportamento?*

Fortemente

Moderatamente

Appena

Per nulla

Non lo so

*24. Secondo te, fino a che punto i bambini con l'epilessia hanno dei problemi relazionali con gli altri bambini?*

Fortemente

Moderatamente

Appena

Per nulla

Non lo so

*25. Rispetto ai loro compagni di classe sani, come dovrebbero essere trattati i bambini con l'epilessia in base alle loro attitudini ed alle loro richieste?*

Allo stesso modo

In modo diverso

Non lo so

*26. In base alla tua esperienza, come si comportano i compagni di classe verso il bambino con epilessia?*

Normalmente

Cercano di aiutarlo

Rendono a marginalizzarlo

Non lo so

*27. Secondo la tua esperienza, attività ricreative e sportive del bambino con l'epilessia dovrebbero essere:*

Normali

Limitate

Non lo so

*28. Quale dei seguenti sport pensi che non dovrebbe assolutamente essere raccomandato ad un bambino con epilessia?*

Calcio

Tennis

Nuoto

Sci

Atletica

Pugilato

Ciclismo

Tutti
