# Supplementary material for: Assessing knowledge and attitudes toward epilepsy among schoolteachers and students: Implications for inclusion and safety in the educational system
Source: PLoS One. 2021 Apr 2;16(4):e0249681. doi: 10.1371/journal.pone.0249681 (PMC8018618; doi:10.1371/journal.pone.0249681)
Supplement: S2 Appendix — (DOCX) [file pone.0249681.s003.docx]

**S2 Appendix**. Students survey questionnaire

***A. General and specific knowledge of epilepsy***

*1. Do you know the disease called “epilepsy”?*

Yes

No

*2. Do you know epilepsy:*

By hearsay

Personal or familial experience

Friends/acquaintances

Medical interviews

Read scientific pamphlets

Participation in courses

*3. Have you ever seen a seizure?*

Classroom

Public place

Home

TV/movies

Never

*4. What is the approximate prevalence of epilepsy in Italy?*

1/10

1/100

1/1.000

1/10.000

1/100.000

1/1.000.000

Don't know

*5. What do you think causes epilepsy*? *(you can check multiple responses)*

Hereditary disease

Birth defect

Viral infection

Head injury

Brain tumor

Stress

*6. What is the age of onset of epilepsy?*

Childhood

Adult

All ages

Don't know

*7. Do you think epilepsy is a form of psychiatric disease?*

Yes

No

Don't know

*8. Do you think epilepsy is treatable with:*

Specific drugs

Neurosurgery

Other methods

Don't know

*9. Do you think epilepsy is a curable illness?*

Yes

No

Don't know

*10. What examination are used to diagnose epilepsy?*

Electroencephalogram (EEG)

X-ray

Computerized tomography/magnetic resonance imaging

Blood tests

Psychological tests

Don’t know

**Section B: Social Impact and attitudes toward epilepsy**

*11. Do you think epilepsy is an important impediment for:*

Driving

Job

Sports

Marriage/having children

Don’t know

12. *In the case of a seizure in class (with loss of consciousness, drop,*

*and spasms of the whole body) what would you do?*

Call an ambulance

Have the person lie down on the ground and wait until the end of

the attack

Place something in the child's mouth

Block the spasms of the limbs

Administer medications endorectally

Would not know what to do

**S2 Appendix**. Students survey questionnaire (Original, Italian language)

***A. Conoscenze generali e specifiche sull’epilessia***

*1. Hai mai sentito parlare di “Epilessia”?*

Si

No

*2. Conosci l’epilessia:*

Per sentito dire

Da esperienze personali/familiari

Da amici/parenti

Da un medico o da personale sanitario

Da articoli scientifici

Da corsi di formazione/universitari

*3. Hai mai visto una crisi epilettica?*

Si, in classe/aula

Si, in un luogo pubblico

Si, a casa

Si, in tv/film

No, mai

*4. Quanto è diffusa l’epilessia in Italia? **

1/10

1/100

1/1.000

1/10.000

1/100.000

1/1.000.000

Non lo so

*5. Secondo te qual è la causa dell’epilessia? (puoi selezionare risposte multiple)*

Malattia ereditaria

Difetti di nascita

Infezioni virali

Traumi alla testa

Tumori cerebrali

Disturbi psicologici/psichiatrici

*6. Qual è l’età di insorgenza dell’epilessia?*

Infanzia

Età adulta

A tutte le età

Non lo so

*7. Pensi che l'epilessia sia una forma di malattia psichiatrica?*

Si

No

Non lo so

*8. Pensi che l’epilessia sia curabile con:*

Farmaci specifici

Neurochirurgia

Altri metodi

Non lo so

*9. Pensi che l’epilessia sia una malattia da cui si può guarire?*

Si

No

Non lo so

*10. Che tipo di esami sono effettuati per fare diagnosi di epilessia?*

Elettroencefalogramma (EEG)

Raggi X

TAC/ Risonanza magnetica

Analisi del sangue

Test psicologici

Non lo so

**Sezione B: Impatto sociale dell’epilessia**

*11. Pensi che l’epilessia sia un limite per:*

Guidare

Lavorare

Fare Sport

Sposarsi/avere figli

Non lo so

12. *In presenza di un attacco epilettico (es. con perdita di conoscenza, caduta e spasmi di tutto il corpo) cosa faresti?*

Chiamo un'ambulanza

Faccio sdraiare la persona a terra e attendo la fine dell'attacco

Metto qualcosa in bocca, per evitare lesioni alla lingua

Somministro farmaci per via rettale

Blocco gli spasmi di braccia e gambe

Non saprei cosa fare
