# Supplementary material for: Assessing knowledge and attitudes toward epilepsy among schoolteachers and students: Implications for inclusion and safety in the educational system
Source: PLoS One. 2021 Apr 2;16(4):e0249681. doi: 10.1371/journal.pone.0249681 (PMC8018618; doi:10.1371/journal.pone.0249681)
Supplement: Attachment — Submitted filename: Response to reviewers.docx [file pone.0249681.s004.docx]

**Reviewers Comments to the Author**

**Reviewer #1:** This was a survey-based study aimed to estimate the knowledge and attitudes toward epilepsy among Italian schoolteachers and students in Italy. Overall, 667 schoolteachers and 672 students were included. The study is very interesting and has practical implication by highlighting the need to improve the general knowledge about seizures and epilepsy in lay population. There are, however, some issues that could be better addressed.

In the abstract, the phrase “Among teachers and students, consider epilepsy a psychiatric disorder (16.8% and 26.5%) or an incurable disease (43.9% and 33%)” should be reworded for greater clarity.

The comparison of the result of the current study with the findings from previous similar reports should be better placed in the discussion rather than result section.

*The sentence in the abstract has been reworded accordingly: “Moreover, some students and teachers believe that epilepsy is a psychiatric disorder (16.8% and 26.5%) or an incurable disease (43.9% and 33%), respectively.”*

*The comparison of the results with previous reports has been reported in the discussion ‘section.*

**Reviewer #2**:

**Comment**

Several studies have evidenced inadequate knowledge about epilepsy and inappropriate seizure management, influencing the quality of life and social inclusion of patients with epilepsy. The experiences of students and teachers reported in this paper underline that epilepsy continues to be persistently mismanaged in the educational system, impacting patients with epilepsy due to inappropriate limitations and epilepsy-related stigma. Therefore, these findings highlight still poor knowledge and attitudes about epilepsy among teachers and students. Improving existing dedicated educational/training interventions are needed.

The manuscript is well written and statistical analysis is correctly addressed. The authors could briefly discuss similar studies in other countries if any.

Difference between out study and other study worldwide have been reported in the discussion section:

*“However, results are in line with the majority of previous studies in Italy [12,13,15] and worldwide [16–20] or slightly better compared to others [9,21]. In the last decade, several studies demonstrated an improved positive attitude during the years despite the continuous lack of knowledge about epilepsy and numerous variables have been implicated, including have a relative with epilepsy or previous experience with a student with epilepsy for teachers [17][22]. Overall, improved knowledge about the disease seem to positively affects attitude toward a person with epilepsy [23], although social preconceptions persist.”*
